# Pelvic Actinomycosis Mimicking Gynecologic Malignancy: CT and Histopathologic Correlation

**DOI:** 10.3390/diagnostics16162586

**Published:** 2026-08-15

**Authors:** Jia Yi Kow, Yi-Wen Su

**Affiliations:** 1 Department of Obstetrics and Gynecology, Dalin Tzu Chi Hospital, Buddhist Tzu Chi Medical Foundation, Chiayi 622, Taiwan; kjy1991@gmail.com; 2 Department of Emergency, Dalin Tzu Chi Hospital, Buddhist Tzu Chi Medical Foundation, Chiayi 622, Taiwan; 3 Department of Nursing, Dalin Tzu Chi Hospital, Buddhist Tzu Chi Medical Foundation, Chiayi 622, Taiwan

**Keywords:** actinomycosis, ovarian neoplasms, intrauterine devices, postmenopause

## Abstract

A healthy 65-year-old postmenopausal woman with a 15-year history of copper intrauterine device use presented to the emergency room with several days of lower abdominal pain and diarrhea. Laboratory testing revealed leukocytosis (white blood cell count, 26.15 × 10^3^/µL) and elevated C-reactive protein (19.81 mg/dL). Contrast-enhanced computed tomography demonstrated multiple cystic and heterogeneous abdominopelvic lesions, including extension across the abdominal wall fascia and muscles, with involvement of the uterus and urinary bladder, raising suspicion for advanced gynecologic malignancy with peritoneal dissemination. Tumor markers were within normal limits. Persistent lower abdominal pain and inflammatory markers without fever and negative blood culture after a week of empirical antibiotics (Flomoxef Sodium 2000 mg) prompted exploratory laparotomy to evaluate for possible ovarian malignancy or abscess. Total hysterectomy, right salpingo-oophorectomy, partial excision of the abdominal abscess, and drainage of the left ovarian abscess were performed. Histopathology later confirmed the diagnosis of diffuse pelvic actinomycosis. The patient recovered after surgical drainage and antibiotics penicillin G for 4 weeks, followed by doxycycline (200 mg) twice daily for 3 months. This case highlights the important radiologic features of pelvic actinomycosis mimicking gynecologic malignancy, particularly in women with prolonged intrauterine device use and the management in refractory pelvic actinomycosis.

**Figure 1:**
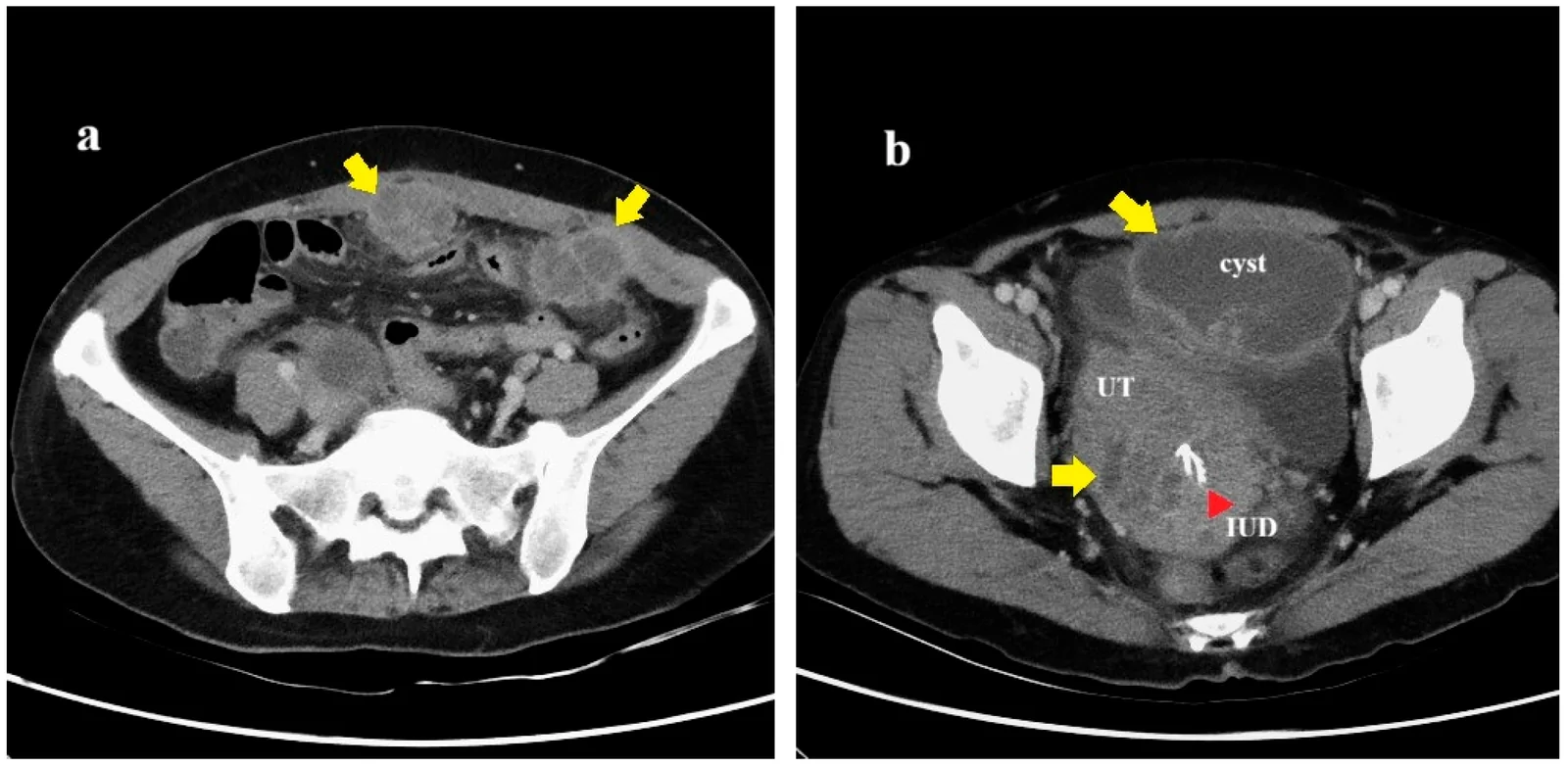
A postmenopausal healthy woman with a history of prolonged copper intrauterine device (IUD) use presented with an extensive cystic lesion involving the abdominal wall, bladder, uterus, and adnexa, initially mimicking a gynecologic malignancy. Transvaginal ultrasonography further demonstrated a cystic lesion adjacent to the bladder, an additional abdominal cystic lesion, and an enlarged, globular uterus containing a myoma. A Pap smear was performed and the IUD was removed. Preoperative contrast-enhanced axial computed tomography (CT) images of the pelvis are shown. (**a**) Multiple cystic lesions within the lower abdominal wall, measuring 37 × 27 mm and 30 × 32 mm, extending across the fascia and muscle layers (arrows). Presence of extensive fat stranding over peritoneum. (**b**) A heterogeneous pelvic cystic lesion measuring 85 × 47 mm located superior to the urinary bladder (arrow), with abscess formation within the uterine myometrium (arrow). The differential diagnosis included uterine, ovarian, and gastrointestinal malignancy, tubo-ovarian abscess and extrapulmonary tuberculosis. All serum tumor marker levels were within normal limits: CA-125, 16.2 U/mL; CA 19-9, 6.20 U/mL; and CEA 2.17 ng/mL. Empirical intravenous antibiotic therapy (Flomoxef Sodium 2000 mg IV every 12 h) was initiated upon admission. After one week of treatment, inflammatory markers remained elevated, with a white blood cell count of 19.75 × 10^3^/µL and a C-reactive protein level of 18.39 mg/dL. Cervical cytology revealed the presence of actinomyces-like organisms. Pelvic actinomycosis was highly suspected. However, due to persistent abdominal pain and leukocytosis and concern of possible malignancy, an exploratory laparotomy was performed. Greenish purulent material and diffuse intestinal adhesions were observed intraoperatively. During the operation, total abdominal hysterectomy, right salpingo-oophorectomy, and partial excision of the abdominal abscess was performed, as well as drainage of the left ovarian abscess, due to extensive adhesion to ureter and colon. Pus and blood cultures subsequently showed negative growth. Actinomycosis is a chronic granulomatous and suppurative infection caused by anaerobic Gram-positive filamentous bacteria of the genus Actinomyces, which normally colonize the oral cavity, gastrointestinal tract, and female genital tract [1]. Although abdominopelvic involvement is uncommon, it is clinically significant because it frequently presents with nonspecific symptoms and infiltrative masses that mimic gynecologic malignancy [1,2,3]. Among the recognized risk factors, prolonged IUD use is the most important risk factor in women with pelvic disease [1,2]. Preoperative diagnosis is challenging because radiologic findings are nonspecific, cultures are frequently negative, and the disease may extend across normal tissue planes [1,3,4]. Although Actinomyces-like organisms on cervical cytology may be a useful indicator in patients using IUDs, this finding alone is neither diagnostic nor predictive of invasive pelvic actinomycosis [5]. Prolonged IUD use is an important clinical indicator, with the disease typically presenting as an extensive inflammatory pelvic mass involving the urinary tract [6,7,8]. Recent studies emphasize that abdominopelvic actinomycosis remains one of the most frequently misdiagnosed chronic infiltrative pelvic infections [9]. The CT images illustrate key CT criteria that may help differentiate pelvic actinomycosis from ovarian malignancy. Distinctive findings supporting pelvic actinomycosis include internal cystic changes (microabscesses) particularly within the uterine myometrium, prominent peritoneal fat stranding, extension across tissue planes into adjacent organs, absence of ascites, and relative absence of significant lymphadenopathy [3,6]. In contrast, typical CT characteristics of ovarian malignancy include diffuse omental caking, peritoneal carcinomatosis with ascites, and unilateral or bilateral ovarian cystic lesions featuring internal septa, solid components, and rich vascularity [10,11]. Integrating these characteristic imaging features may facilitate a more confident pre-operative diagnosis and narrow the differential diagnosis.

**Figure 2:**
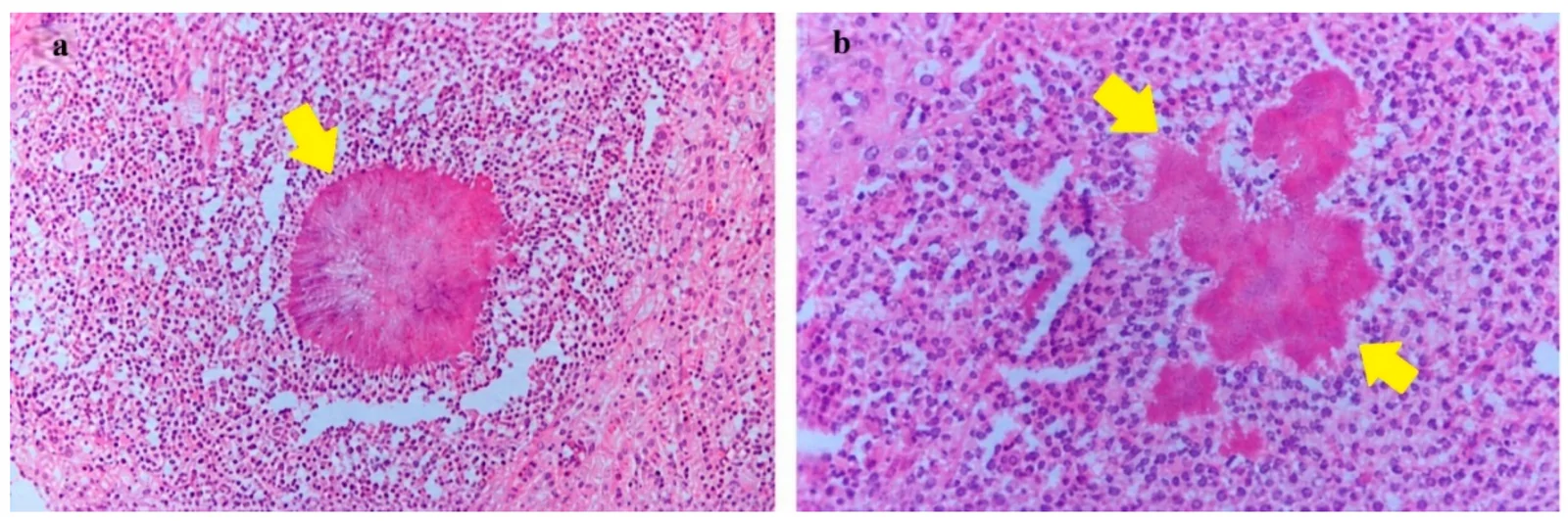
Histopathological examination revealed sulfur granules and Actinomyces-like organisms involving the abdominal wall muscle and fascia, muscularis propria of the urinary bladder, uterine myometrium, right fallopian tube, and ovary. Histopathologic findings of the resected abdominopelvic lesion are shown. (**a**) Hematoxylin and eosin (H&E) staining demonstrated an eosinophilic actinomycotic colony (sulfur granule) surrounded by dense neutrophilic infiltrates and abscess formation (arrow). (**b**) H&E staining shows irregular eosinophilic club-shaped material with surrounding inflammatory cell infiltrates, consistent with the Splendore–Hoeppli phenomenon (arrow). There was no evidence of malignancy. The patient subsequently started intravenous penicillin G (20Mu) every 4 h followed by oral doxycycline (100 mg) twice daily for at least 3 months. The patient’s postoperative course was complicated by a small bowel leak, which resolved with conservative management. The patient was discharged 3 weeks after surgery. At the 3-month follow-up visit, sonography and a CT scan showed the abdominopelvic abscess had resolved, with no residual pelvic cystic lesions identified. At the 6-month follow-up visit, all symptoms had resolved, including the resolution of right hydronephrosis. In our patient, the final diagnosis was established by histopathological examination, revealing the characteristic sulfur granules and Splendore–Hoeppli phenomenon, which remains the reliable diagnostic basis for actinomycosis other than microbiological confirmation [1,12]. The prognosis of pelvic actinomycosis is generally favorable when the disease is recognized early and treated with prolonged penicillin-based antimicrobial therapy [1,2,8,9]. From a preventive perspective, prolonged IUD retention should be avoided, and women using an IUD should receive regular gynecologic follow-up. Early evaluation and timely removal of a long-standing IUD should be included in differential diagnosis in women with menopause, new-onset pelvic pain, abnormal discharge, or pelvic mass is detected to avoid unnecessary radical surgery when preoperative diagnosis is possible [1,5,8].

## Data Availability

The original contributions presented in this study are included in the article. Further inquiries can be directed to the corresponding authors.

## Abbreviations

The following abbreviations are used in this manuscript:

H&E	Hematoxylin and Eosin
IUD	Intrauterine device
CT	Computed tomography
CEA	Carcinoembryonic antigen
CA	Cancer antigen
